# A Green Chemical Approach for Iodination of Pyrimidine Derivatives by Mechanical Grinding under Solvent-Free Conditions

**DOI:** 10.3390/molecules27196386

**Published:** 2022-09-27

**Authors:** Thananjeyan Balasubramaniyam, Byeong-Seon Kim, Badvel Pallavi, Ho-Seong Jin, Sung Kuk Kim, Joon-Hwa Lee

**Affiliations:** 1 Department of Chemistry and RINS, Gyeongsang National University, Jinju 52828, Korea; 2 Department of Chemistry Education and RINS, Gyeongsang National University, Jinju 52828, Korea

**Keywords:** green synthesis, mechanochemistry, nitrate salts, pyrimidine derivatives

## Abstract

The iodination of pyrimidines is usually carried out by using toxic reagents under acidic conditions, such as with sulfuric acid and nitric acid. To avoid toxic reagents, we developed a simple and eco-friendly approach for the iodination of pyrimidine derivatives under solvent-free conditions using solid iodine and AgNO_3_ as an electrophilic iodinating reagent. The advantages of this method are the relatively short reaction time (20–30 min), simple set-up procedure, high yields (70–98%), and environmentally friendly reaction conditions. Our novel approach for the iodination of pyrimidines, as well as a variety of their derivatives, will contribute to the development of nucleobase-related drug candidates.

## 1. Introduction

Many studies on modified nucleobases have reported that C5-halogeneted (iodo and bromo) pyrimidine is highly active in medicinal usage, and is widely utilized for therapeutic purposes. In particular, iodinated uridine (C5-iodo-2′-deoxyuridine) is widely used as an antiviral drug [1,2]. Iodinated nucleotides are essential precursors for various functional group transformations [3,4]. Therefore, developing a convenient procedure for the iodination of pyrimidines is an important field in synthetic bioorganic chemistry.

**Scheme 1:**
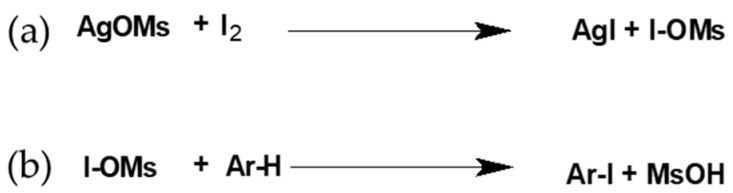
Chemical reaction scheme for the synthesis of aromatic iodides.

In the production of a wide variety of pharmaceutical and bioactive materials, aromatic iodides, including iodinated pyrimidines, play an essential role as intermediates [5,6]. Electrophilic aromatic substitutions are one of the most widely used synthetic methods to create C-I bonds in aromatic iodides [7,8]. In the case of the electron-deficient arenes and heterocycles, they can be iodinated through the combination of molecular iodine (I_2_) and electrophilic iodinating reagents, such as silver methylsulfonate (AgOMs), as follows (Scheme 1) [9]:

Ar-H and Ar-I indicate the aromatic molecules and aromatic iodide, respectively. Pyrimidines, which are one type of heterocycles, and their derivatives are also able to be iodinated through similar methods.

The electrophilic iodination of aromatic substrates is quite difficult because iodine has weak reactivity. Thus, this reaction can only occur under harsh reaction conditions such as acidic conditions using nitric acid, acetic acid, or sulfuric acid, or using strong oxidative agents as iodination sources. Environmentally friendly methods for the iodination of pyrimidines have not yet been reported. 

**Scheme 2:**
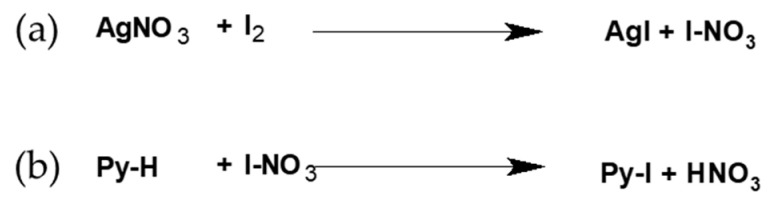
Chemical reaction scheme for the synthesis of 5-iodo-pyrimidine derivatives.

**Figure 1:**
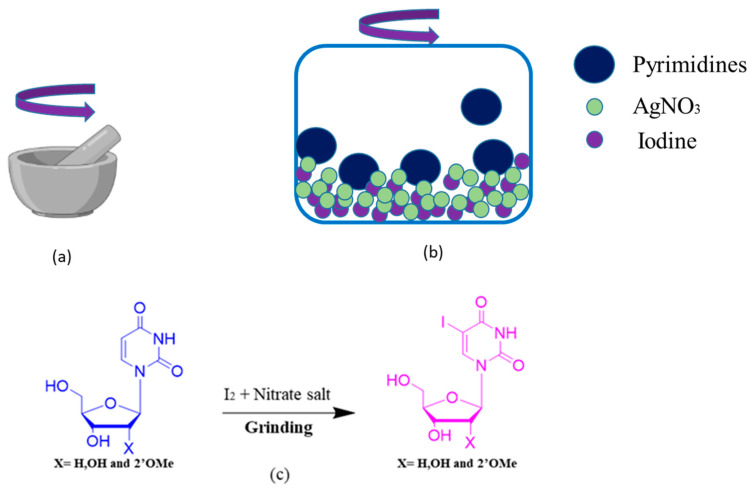
Schematic representations of the mechanical grinding reaction. (**a**) Grinding with a mortar and pestle (“grindstone” activation); (**b**) high-speed vibration milling in a mixer mill (activation of the compound); (**c**) C5 iodination of pyrimidine derivatives under solvent-free conditions.

We developed solvent-free mechanochemistry methods for the iodination of pyrimidines, namely uracil and cytosine, as well as their derivatives, which have advantages in terms of generating less pollution and lower costs. We developed a procedure for iodination at the C5 position of pyrimidines via a solid-state reaction, performed by grinding all of the reaction mixtures (Figure 1). We performed the reactions using molecular iodine (I_2_) and various nitrate/nitrite salts (AgNO_3_, NaNO_3_, and NaNO_2_) as follows (Scheme 2):

In this study, direct iodination at the C5 position of the pyrimidines using nitrate salts and iodine was performed under solvent-free conditions at room temperature (Figure 1). The combination of iodine and nitrate salts was found to be the best reagent for the regioselective iodination reactions shown in Figure 1c. The C5-iodo pyrimidine derivatives were confirmed and identified by ^1^H and ^13^C NMR and ESI mass spectroscopy. This study suggests an environmentally friendly approach for the iodination of pyrimidine derivatives that will contribute toward the development of nucleobase-related drug candidates.

## 2. Results and Discussion

### 2.1. Optimization of Iodination Reactions of Uracil and Cytosine

A previous study reported that AgNO_3_ was found to give better results than other nitrate salts for the simple aromatic substrates [7]. Here, AgNO_3_ acted as a Lewis acid and generated nitryl iodide in situ by reaction of silver nitrate with iodine (Scheme 2). Although the grinding reactions were carried out in a solvent-free environment, a few drops of acetonitrile were used for easier grinding. Interestingly, this gentle grinding method was effective for C5 iodination under solid iodine and silver nitrate conditions. Furthermore, the common reaction byproduct was silver iodide (Scheme 2) [7,10]. 

Our initial optimization of the iodination reaction used uracil (U) as a model system. The optimization was tested with AgNO_3_ (0.5 Equiv.), molecular iodine (I_2_) (1.2 Equiv.), and 0.1 mmol uracil, based on a previous study [11], under several different reaction times. During optimization, the relative molar ratio of AgNO_3_ compared to uracil was changed in the range of 0.5~2.5 (Table 1). At 0.5 Equiv. of AgNO_3_, the reaction at 25 °C produced 5-iodo-uracil (5I-U) in 38% yield (10 min) and 63% yield (30 min) (Table 1). When the amount of AgNO_3_ was increased to 2.0 Equiv., 90% of 5I-U was detected after 30 min (Table 1). However, the reaction yield did not increase, even though AgNO_3_ was increased up to 2.5 Equiv. (Table 1). Thus, the optimal amount of AgNO_3_ in this reaction is 2.0 Equiv. compared to uracil (Table 1).

Similarly, the optimization of iodination of cytosine was also performed. As shown in Table 2, 2.0 Equiv. of AgNO_3_ was required for optimal reaction in the iodination of cytosine.

### 2.2. Iodination Reaction Using AgNO_3_

#### 2.2.1. Iodination of Uracil and Uridine Derivatives

The iodination of uridine (rU) and 2′-deoxyuridine (dU) was performed with 2.0 Equiv. of AgNO_3_ for 25 min (optimized conditions). The reaction mixture was filtered, washed with methanol, and purified with silica-gel column chromatography. The 5-iodo-uridine derivative products, 5-iodo-uridine (5I-rU) and 5-iodo-2′-deoxyuridine (5I-dU), were identified by ^1^H NMR spectra, in which the H5 signals disappeared (Supplementary Figures S2 and S3). The reaction yields for the 5I-rU and 5I-dU were 83% and 86%, respectively (Table 3).

We also performed the iodination reaction on the sugar-modified 2′-OMe-uridine (mU). Interestingly, the reaction to 5-iodo-2′-OMe-uridine (5I-mU) was completed within 15 min with a 98% yield (Table 3).

#### 2.2.2. Iodination of Cytosine and Cytidine Derivatives

Next, the iodination of cytidine (rC), 2′-deoxycytidine (dC), and 2′-OMe-cytidine (mC) was also performed with 2.0 Equiv. of AgNO_3_ for 30 min (optimized conditions) to produce 5-iodo-cytidine (5I-rC), 5-iodo-2′-deoxycytidine (5I-dC), and 5-iodo-2′-OMe-cytidine (5I-mC), respectively. The iodination products, 5I-rC, 5I-dC, and 5I-mC, were identified by the ^1^H and ^13^C -NMR spectra (Supplementary Figures S6–S8). Interestingly, the 5I-rC was produced with a yield of only 59%, which was significantly lower than the other cytidine derivatives (Table 3).

### 2.3. Iodination Reaction Using Ag_2_SO_4_, NaNO_3_, and NaNO_2_

In order to clarify the salt effect on the solvent-free iodination of pyrimidine derivatives, we also performed the same reaction with Ag_2_SO_4_, NaNO_3_, and NaNO_2_ as the Lewis acid to generate nitryl iodide.

In the case of uracil, the reaction yield to 5-iodo-uracil was 73% when Ag_2_SO_4_ was used (Table 3). However, the reaction with NaNO_3_ and NaNO_2_ led to reaction yields of only 33% and 38%, respectively (Table 3). A similar result was observed for the iodination of uridine (Table 3). Surprisingly, when Ag_2_SO_4_, NaNO_3_, and NaNO_2_ were used, only <50% of dU and mU could be converted to 5I-dU and 5I-mU, respectively (Table 3).

In the case of cytidine derivatives, all iodination reactions with Ag_2_SO_4_, NaNO_3_, and NaNO_2_ showed reaction yields lower than 35%, except the iodination of mU with Ag_2_SO_4_ (Table 3). The iodination of mU with Ag_2_SO_4_ had 80% yield (Table 3).

The synthetic strategy for the electrophilic iodination of pyrimidine was an electrophilic reaction of the reactive iodine species produced by nitrate salt. Table 3 summarizes the direct iodination and the oxidative iodination required for I^+^ species, which were generated by an electrophilic iodinating reagent (Ag_2_SO_4_), in comparison to the other metal catalysts (NaNO_3_ and NaNO_2_) [12,13,14]. The reactivity was different depending on each pyrimidine substrate. Based on the obtained results, we can conclude that the order of the reactivity of the nitrate salts is AgNO_3_ >> Ag_2_SO_4_ >> NaNO_3_ >> NaNO_2_. 

It has been reported that electrophilic reactions can be catalyzed by metal-based salts [15,16]. Silver-containing salts can more efficiently catalyze this reaction [17]. Our results revealed that AgNO_3_ is a better catalyst for the direct iodination reaction compared to Ag_2_SO_4_, even though both contain the Ag^+^ ion. In the presence of oxygen, NaNO_3_ and NaNO_2_ can also promote this reaction, although their efficiencies were slightly lower than the Ag^+^-based catalysts. In addition, NaNO_2_ showed higher efficiency than NaNO_3_, because it can be converted to nitrogen oxide [12,18].

## 3. Materials and Methods

### 3.1. General Information for Experiments

All the reagents and solvents were purchased from local commercial suppliers. The elemental iodine and nitrate salts were purchased from Dungsun and used without further purification. The ^1^H NMR spectra were recorded on a Bruker 300-MHz instrument with DMSO as the solvent and TMS as the internal standard (TMS, δ = 0.00 ppm). The ^1^^3^C NMR spectra were recorded on a Bruker 500 MHz instrument with DMSO as the solvent. All the products were identified by comparison of their spectral and physical data with those of the known sample. The progress of the reaction and the purity of the products were checked by thin-layer chromatography (TLC) on silica gel 60 F254-coated TLC plates (Merck KGaA, Darmstadt, Germany) and visualized by short-UV light at 254 nm. The coupling constants (*J*) were reported in Hz. Peak multiplicity was indicated as follows: s, singlet; d, doublet; t, triplet; q, quartet; m, multiplet; and dd, doublet of the doublet. Mass spectra were obtained by an LCQ Fleet ion trap mass spectrometer using positive-ion (ESI+) and negative-ion (ESI–) mode electrospray ionizations (ThermoScientific; Qual Browser software, version 2.0.7, Thermo Fischer Scientific, Waltham, MA, USA). An amount of 20 μL of the sample was diluted with HPLC grade methanol solution for analysis. HPLC separation with UV was performed on a 1290 infinity UHPLC system (Agilent technology, Waldbroen, Germany) with a 1μL detection cell and a 20 μL sample loop. The chromatographic column was carried out using a Waters µBondapak C18 Column, 10 µm, 7.8 mm × 300 mm. 

### 3.2. Synthesis of 5-Iodo Pyrimidine Derivatives

Uracil and cytosine (4.4 mmol), iodine in the solid state (1.12 mmol), nitrate salts (9 mmol), and 2–4 drops of acetonitrile were mixed in a mortar. The reaction mixture was ground together in a mortar using a pestle to generate a violet-colored tacky solid within 20–30 min. The reaction proceeded exothermically (indicated by an increase in temperature of 20–35 °C). After the reaction was completed (as confirmed by TLC), a saturated solution of sodium thiosulfate (5 mL) was added to remove the unreacted iodine, and the remaining solid was separated out. The resulting solid was washed with a saturated solution of sodium thiosulfate (5 mL), filtered, and then finally washed with cold water. The crude products were purified by silica gel column chromatography (solvent: CH_2_Cl_2_: MeOH). 

### 3.3. Spectroscopic Details of C5-Iodo Pyrimidine Derivatives

#### 3.3.1. 5-Iodo-Uracil (5I-U)

5I-U was obtained according to the general procedure as an off-white solid (0.96 g, 90% yield). ^1^H NMR (300 MHz, DMSO-*d*_6_) δ 11.54 (s, 1H), 7.97 (s, 4H). ^13^C NMR (500 MHz, DMSO-*d*_6_) δ 161.96, 151.69, 147.42, 68.05. MP (259 °C) [15]. HRMS (ESI) calcd. for C_4_H_3_IN_2_O_2_ [M + H]^+^ 237.9845, found at 237.9167.

#### 3.3.2. 5-Iodo-Uridine (5I-rU)

5I-rU was obtained according to the general procedure as an off-white solid (0.63 g, 83% yield). ^1^H NMR (300 MHz, DMSO-*d*_6_) δ 11.68 (s, 1H), 8.48 (s, 1H), 5.72 (d, *J* = 4.6 Hz, 1H), 4.01 (dt, *J* = 16.7, 4.8 Hz, 2H), 3.86 (dt, *J* = 4.8, 2.8 Hz, 1H), 3.68 (dd, *J* = 12.1, 2.9 Hz, 1H), 3.57 (dd, *J* = 12.0, 2.7 Hz, 1H), 3.17 (s, 0H). 2.5.7 (s, 0H). ^13^C NMR (500 MHz, DMSO-*d*_6_) δ 160.96, 150.84, 145.61, 88.76, 85.19, 74.40, 69.85, 69.76, 60.66, 49.08. MP (206–208 °C) [19]. HRMS (ESI) calcd. for C_9_H_11_IN_2_O_6_ [M + H]^+^ 370.0995, found at 369.1667.

#### 3.3.3. 5-Iodo-2′-Deoxyuridine (5I-dU)

5I-dU was obtained according to the general procedure as an off-white solid (0.67 g, 86% yield). ^1^H NMR (300 MHz, DMSO-*d*_6_) δ 11.66 (s, 1H), 8.40 (s, 1H), 6.10 (t, *J* = 6.5 Hz, 1H), 4.29–4.19 (m, 1H), 3.79 (q, *J* = 3.2 Hz, 1H), 3.65–3.51 (m, 2H), 2.20–2.04 (m, 2H). ^13^C NMR (500 MHz, DMSO-*d*_6_) δ 160.97, 150.58, 145.53, 88.00, 85.13, 70.48, 69.73, 61.30. MP (180–183 °C) [19]. HRMS (ESI) calcd. for C_9_H_11_IN_2_O_5_ [M + H]^+^ 354.1005, found at 353.9707.

#### 3.3.4. 5-Iodo-2′-OMe-Uridine (5I-mU)

5I-mU was obtained according to the general procedure as an off-white solid (0.73 g, 98% yield). ^1^H NMR (300 MHz, DMSO-*d*_6_) δ 11.69 (s, 1H), 8.53 (s, 1H), 5.78 (d, *J* = 3.9 Hz, 1H), 4.11 (t, *J* = 5.2 Hz, 1H), 3.85 (dd, *J* = 5.6, 2.7 Hz, 1H), 3.79 (t, *J* = 4.5 Hz, 1H), 3.68 (d, *J* = 2.7 Hz, 1H), 3.57 (dd, *J* = 12.1, 2.5 Hz, 1H), 3.38 (s, 4H). ^13^C NMR (500 MHz, DMSO-*d*_6_) δ 160.97, 150.60, 145.33, 87.02, 85.27, 83.51, 69.78, 68.28, 60.14, 58.05. MP (149–151 °C) [19]. HRMS (ESI) calcd. for C_10_H_13_IN_2_O_6_ [M + H]^+^ 384.1265, found at 383.9813.

#### 3.3.5. 5-Iodo-Cytosine (5I-C)

5I-C was obtained according to the general procedure as an off-white solid (0.95 g, 90% yield). ^1^H NMR (300 MHz, DMSO-*d*_6_) δ 11.55 (s, 4H), 7.97 (s, 5H), 5.87 (d, *J* = 7.2 Hz, 1H). ^13^C NMR (500 MHz, DMSO) δ 164.66, 154.87, 150.94, 145.94, 93.47, 56.13. MP (149–151 °C) [19]. HRMS (ESI) calcd. for C_4_H_4_ IN_3_O [M + H]^+^ 237.0005, found at 236.9394.

#### 3.3.6. 5-Iodo-Cytidine (5I-rC)

5I-rC was obtained according to the general procedure as an off-white solid (0.45 g, 59% yield). ^1^H NMR (500 MHz, DMSO-*d*_6_) δ 9.53 (s, 1H), 8.53–8.05 (m, 2H), 6.10 (d, *J* = 7.9 Hz, 1H), 5.72 (d, *J* = 3.6 Hz, 1H), 4.06 (t, *J* = 4.2 Hz, 1H), 3.98 (s, 0H), 3.96–3.89 (m, 1H), 3.72 (dd, *J* = 12.3, 2.9 Hz, 1H), 3.60 (dd, *J* = 12.3, 3.0 Hz, 1H). ^13^C NMR (500 MHz, DMSO-*d*_6_) δ 159.52, 147.86, 144.95, 94.27, 89.98, 85.20, 74.59, 69.32, 60.43. HRMS (ESI) calcd. for C_9_H_12_IN_3_O_5_ [M + H]^+^ 369.1155, found at 369.1667. MP (225 °C) [19].

#### 3.3.7. 5-Iodo-2′-Deoxycytidine (5I-dC)

5I-dC was obtained according to the general procedure as an off-white solid (0.61 g, 79% yield). ^1^H NMR (300 MHz, DMSO-*d*_6_) δ 9.42 (s, 30H), 8.23 (s, 2H), 4.22 (s, 1H), 3.86 (s, 1H), 2.14 (d, *J* = 6.4 Hz, 1H). ^13^C NMR (500 MHz, DMSO-*d*_6_) δ 159.54, 147.70, 144.96, 94.22, 88.50, 86.30, 70.22, 61.26. MP (175–177 °C) [19]. HRMS (ESI) calcd. for C_9_H_12_IN_3_O_4_ [M + H]^+^ 353.1165, found at 352.9867.

#### 3.3.8. 5-Iodo 2′-OMe-Cytidine (5I-mC)

5I-mC was obtained according to the general procedure as an off-white solid (0.71 g, 95% yield). ^1^H NMR (300 MHz, DMSO-*d*_6_) δ 11.48 (s, 1H), 8.54 (s, 1H), 5.79 (dd, *J* = 7.2, 3.0 Hz, 1H), 4.07 (dd, *J* = 6.8, 4.9 Hz, 1H), 3.57 (dd, *J* = 12.1, 2.3 Hz, 1H). ^13^C NMR (500 MHz, DMSO-*d*_6_) δ 167.57, 159.99, 150.48, 148.16, 88.23, 83.41, 69.03, 67.38, 61.70, 55.63, 49.08. HRMS (ESI) calcd. for C_10_H_14_IN_3_O_5_ [M + H]^+^ 383.1425, found at 383.0833.

## 4. Conclusions

In conclusion, this study identified a convenient and highly effective method for the solid-state iodination of C5-pyrimidines using iodine (the grinding method). The advantages of this method are the relatively short reaction time (20–30 min), simple set-up procedure, high yields (70–98%), and environmentally friendly reaction conditions that do not require the use of any additional reagents or solvents. Detailed information on the solid-state iodination reaction at the C5 position in various pyrimidine derivatives was also reported. We believe that this method will be helpful in future nucleobase-related drug design and related research.

## Figures and Tables

**Table 1:** Optimization of the iodination of uracil at room temperature ^1^.

Molar Ratio of AgNO_3_^2^		Yield (%) ^3^	
	10 min	20 min	30 min
0.5	38	50	63
1.0	38	63	68
1.5	50	64	77
2.0	65	86	90 ^4^
2.5	68	83	90

^1^ The relative molar ratio of I_2_ compared to the substrate was 1.2. ^2^ The relative molar ratio of AgNO_3_ compared to the substrate. ^3^ Reaction yield was determined from the weight of the final product. ^4^ Optimized condition.

**Table 2:** Optimization of the iodination of cytosine at room temperature ^1^.

Molar Ratio of AgNO_3_^2^		Yield (%) ^3^	
	10 min	20 min	30 min
0.5	30	55	60
1.0	40	60	65
1.5	55	63	70
2.0	73	80	90 ^4^
2.5	70	81	90

^1^ The relative molar ratio of I_2_ compared to the substrate was 1.2. ^2^ The relative molar ratio of AgNO_3_ compared to the substrate. ^3^ Reaction yield was determined from the weight of the final product. ^4^ Optimized condition.

**Table 3:** Summary of the iodination at the C5 position of pyrimidine derivatives with metal catalysts at room temperature ^1^.

Substrate	Product	Structure of Product	Molar Ratio ^2^	Yield (%) ^3^			
				AgNO_3_	Ag_2_SO_4_	NaNO_3_	NaNO_2_
Uracil (U)	5-iodo-uracil (5I-U)	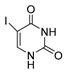	2.0	90	73	33	38
Uridine (rU)	5-iodo-uridine (5I-rU)	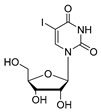	2.0	83	70	50	34
Deoxyuridine (dU)	5-iodo-2′-deoxyuridine (5I-dU)	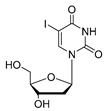	2.0	86	9	18	14
2′-OMe-uridine (mU)	5-iodo-2′-OMe-uridine (5I-mU)	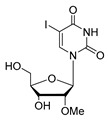	2.0	98	36	6	48
Cytosine (C)	5-iodo-cytosine (5I-C)	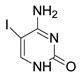	2.0	90	28	29	18
Cytidine (rC)	5-iodo-cytidine (5I-rC)	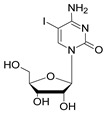	2.0	59	5	4	19
Deoxycytidine (dC)	5-iodo-2′-deoxycytidine (5I-dC)	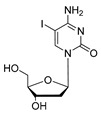	2.0	79	10	7	62
2′-OMe-cytidine	5-iodo-2′-OMe-cytidine (5I-mC)	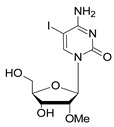	2.0	95	80	32	10

^1^ The relative molar ratio of I_2_ compared to the substrate was 1.2. ^2^ The relative molar ratio of the salt compared to the substrate. ^3^ Reaction yield was determined from the weight of the final product.

## Data Availability

The data presented in this study are available on request from the corresponding authors.

## Supplementary Materials

The following supporting information can be downloaded at: https://www.mdpi.com/article/10.3390/molecules27196386/s1, Figures S1–S8: ^1^H, ^13^C, and ESI-Ms spectra of the 5-iodo pyrimidine derivatives and Figure S9: TLC Rf values of the 5-iodo pyrimidine derivatives.

## Sample Availability

Samples of all compounds are available from the corresponding authors.
