# Supplementary material for: A Green Chemical Approach for Iodination of Pyrimidine Derivatives by Mechanical Grinding under Solvent-Free Conditions
Source: Molecules. 2022 Sep 27;27(19):6386. doi: 10.3390/molecules27196386 (PMC9571335; doi:10.3390/molecules27196386)
Supplement: Supplementary file 1 [file molecules-27-06386-s001.zip › molecules-1893458-supplementary.pdf]

Supporting Information for A green chemistry approach for iodination of pyrimidine derivatives by mechanical grinding under solvent-free conditions

Thananjeyan Balasubramaniyam[1,#], Byeong-Seon Kim[2,#], Badvel Pallavi[2], Ho-seong Jin[1], Sung Kuk Kim[1,*] and Joon-Hwa Lee[1,*]

(A)

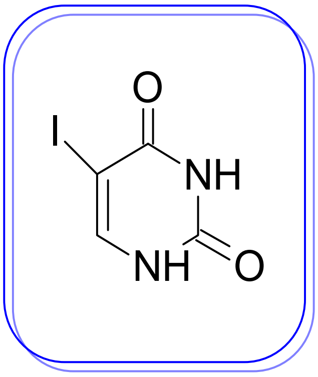

I
O
NH
NH
O

(B)

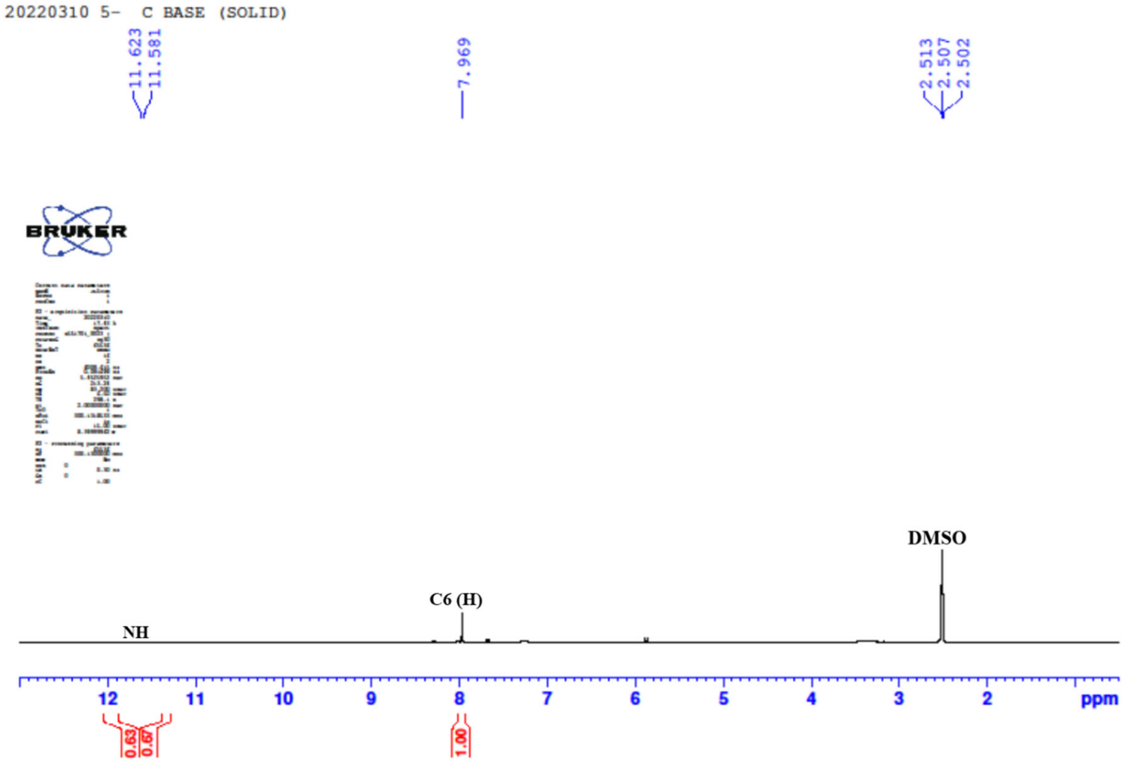

20220310 5- C BASE (SOLID)
11.623
11.581
7.969
2.513
2.507
2.502
BRUKER
DMSO
C6 (H)
NH
12
11
10
9
8
7
6
5
4
3
2
ppm
0.63
0.67
1.00

(C)

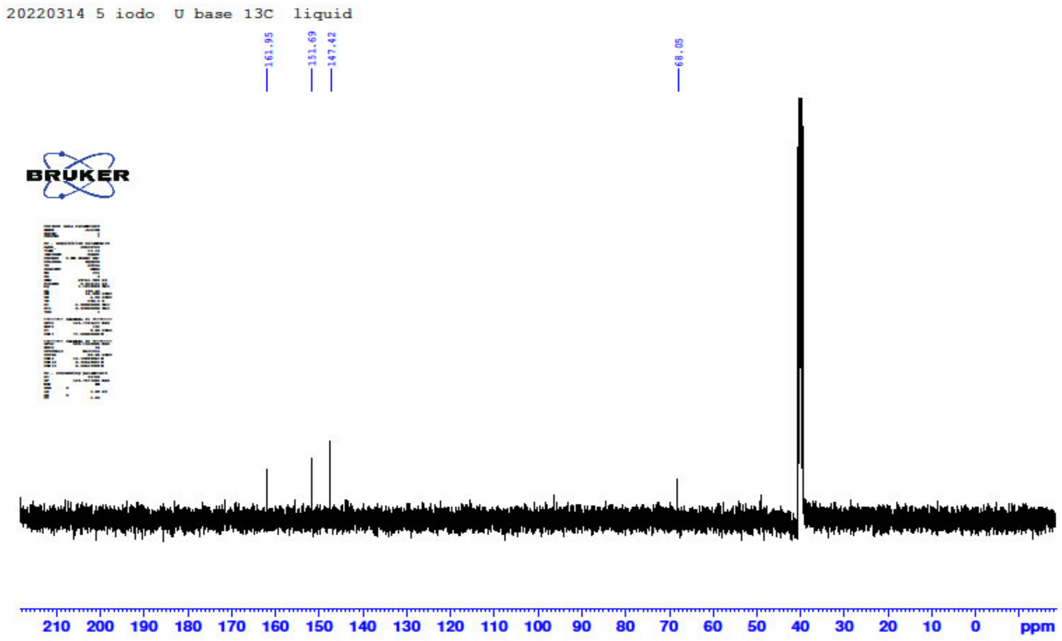

(D)

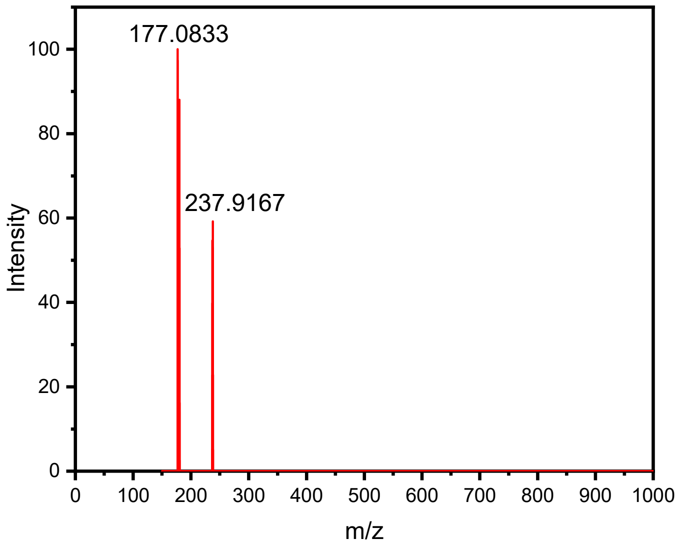

Figure S1. (A) Chemical structure and (B) ^{1}H NMR, (C) ^{13}C NMR, and (D) ESI-Mass spectra of 5-iodo-uracil (5I-U).

(A)

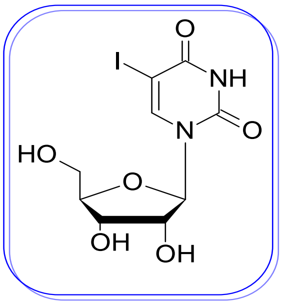

O
I
NH
N
O
HO
O
OH
OH

(B)

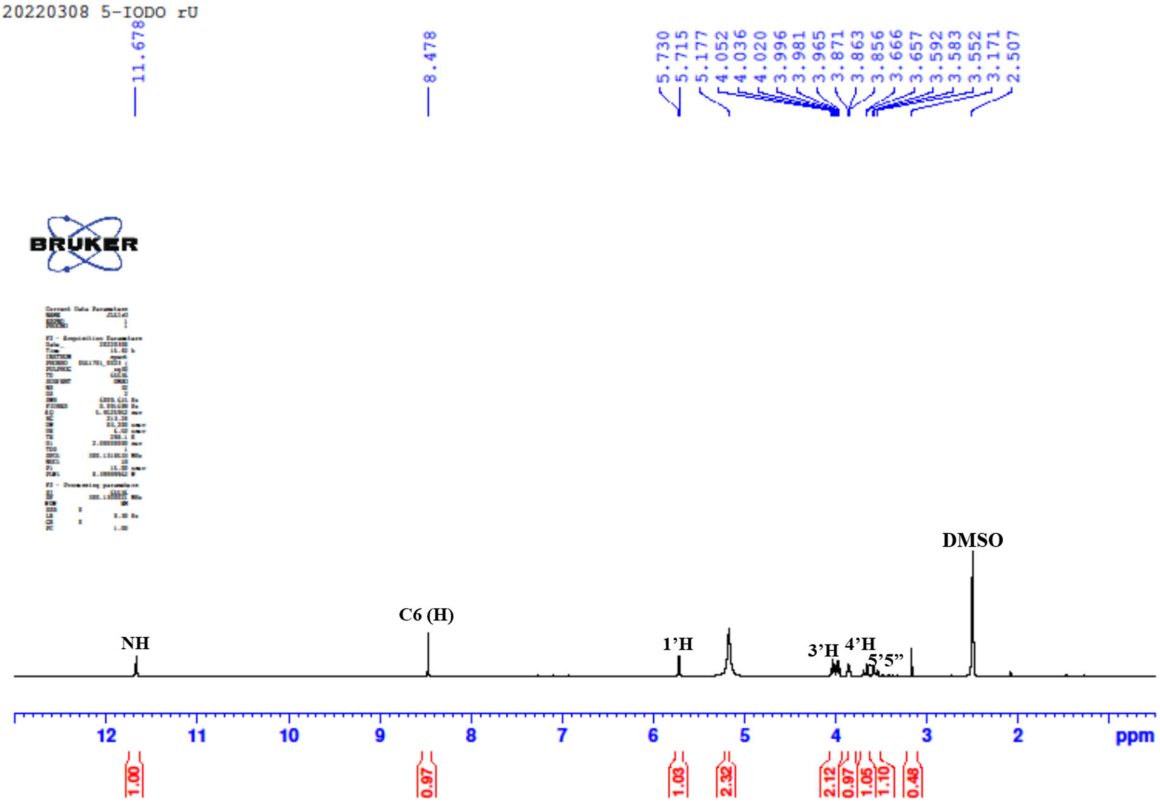

20220308 5-IODO rU
11.678
8.478
5.730
5.715
5.177
4.052
4.036
4.020
3.996
3.981
3.965
3.871
3.863
3.856
3.666
3.657
3.592
3.583
3.552
3.171
2.507
BRUKER
NH
C6 (H)
1'H
3'H
4'H
5'5"
DMSO
12
11
10
9
8
7
6
5
4
3
2
ppm
1.00
0.97
1.03
2.32
2.12
0.97
1.05
1.10
0.48

(C)

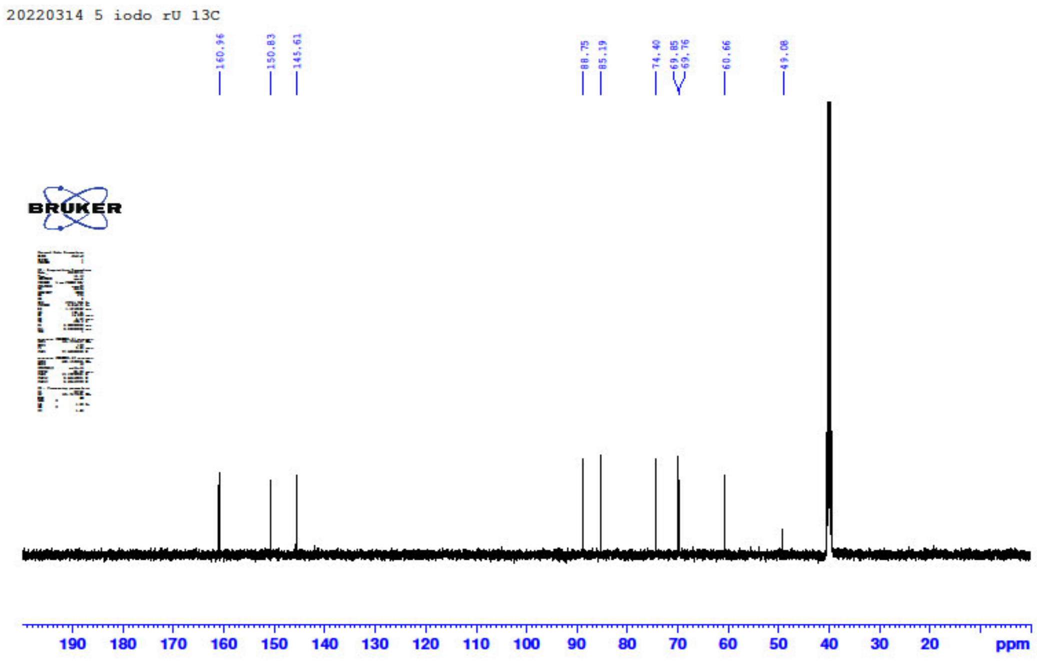

(D)

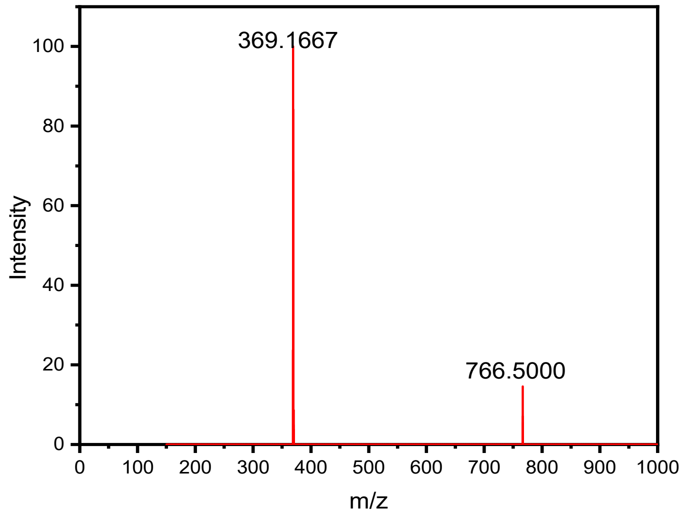

Figure S2. (A) Chemical structure and (B) ^{1}H NMR, (C) ^{13}C NMR, and (D) ESI-Mass spectra of 5-iodo-uridine (5I-rU).

(A)

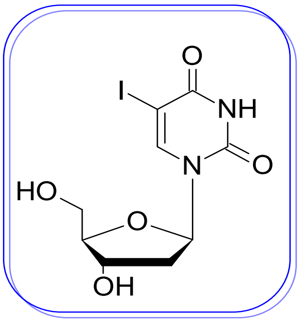

O
I
NH
N
O
HO
O
OH

(B)

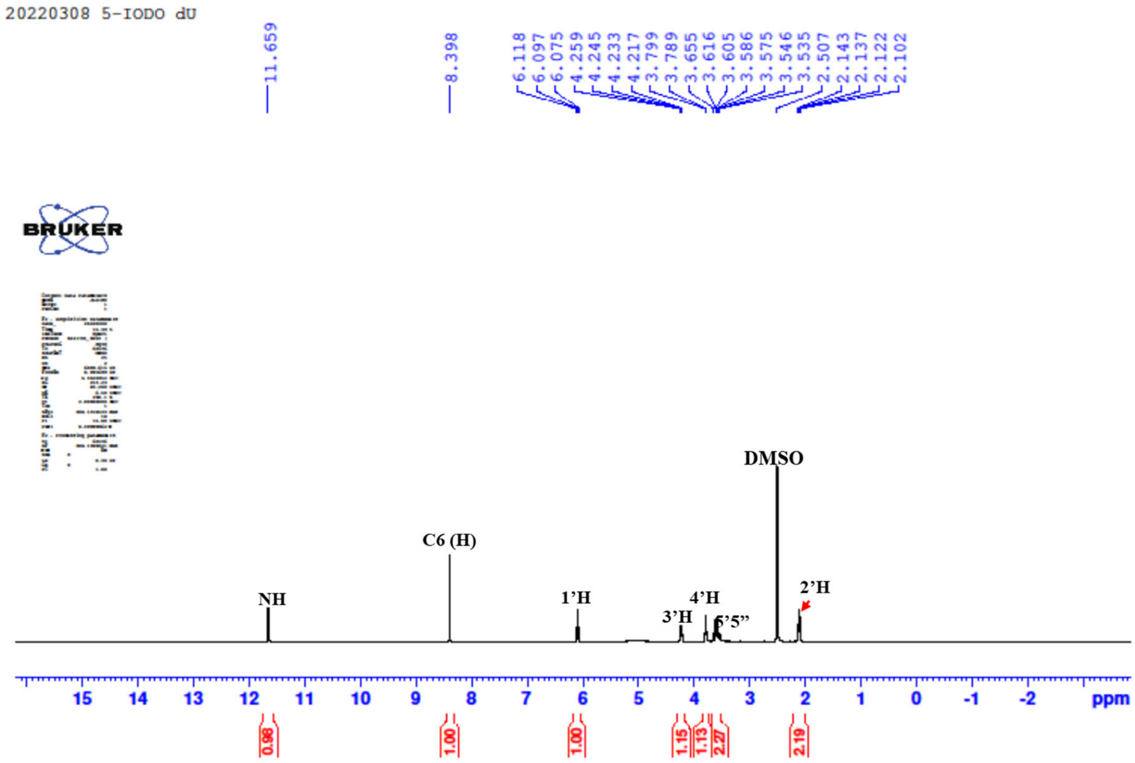

20220308 5-IODO dU
11.659
8.398
6.118
6.097
6.075
4.259
4.245
4.233
4.217
3.799
3.789
3.655
3.616
3.605
3.586
3.575
3.546
3.535
2.507
2.143
2.137
2.122
2.102
BRUKER
DMSO
C6 (H)
NH
1'H
4'H
3'H
5'5"
2'H
15
14
13
12
11
10
9
8
7
6
5
4
3
2
1
0
-1
-2
ppm
0.98
1.00
1.00
1.15
1.13
2.27
2.19

(C)

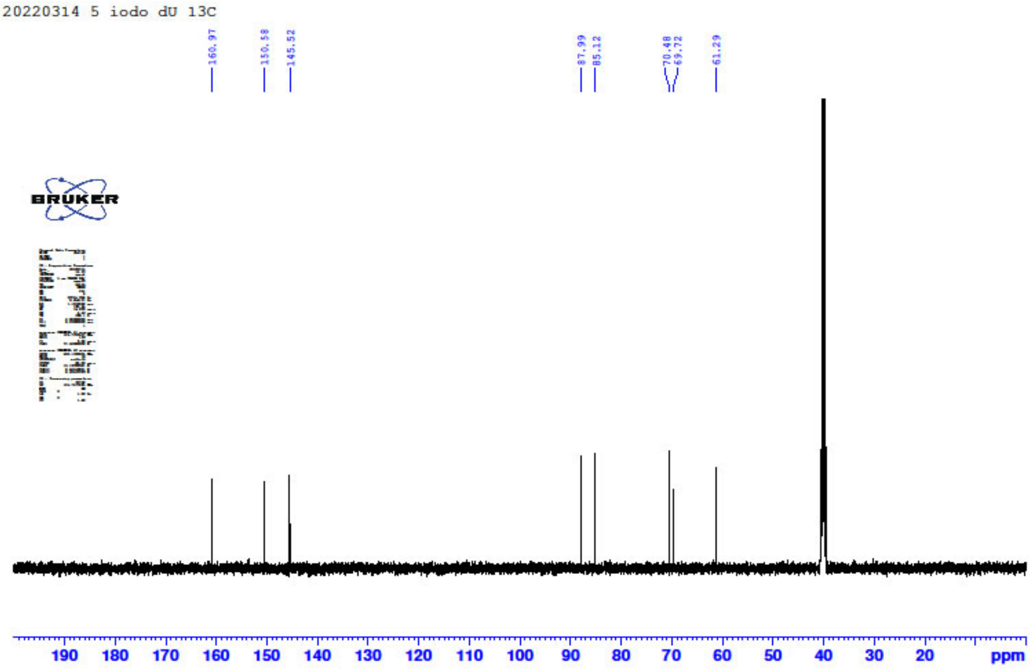

(D)

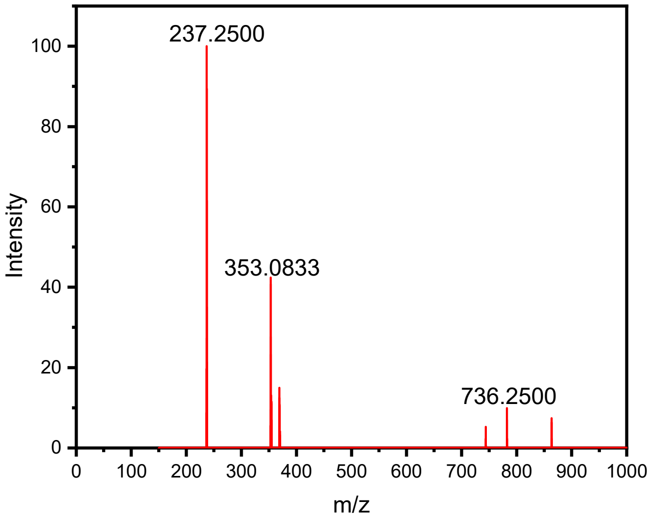

Figure S3. (A) Chemical structure and (B) ^{1}H NMR, (C) ^{13}C NMR, and (D) ESI-Mass spectra of 5-iodo-2′-deoxyuridine (5I-dU).

(A)

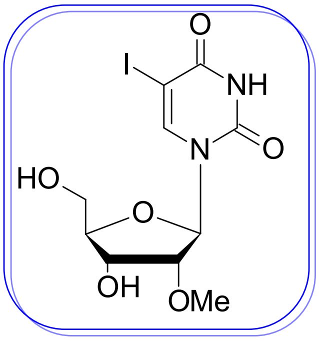
O
I
NH
N
O
HO
O
OH
OMe

(B)

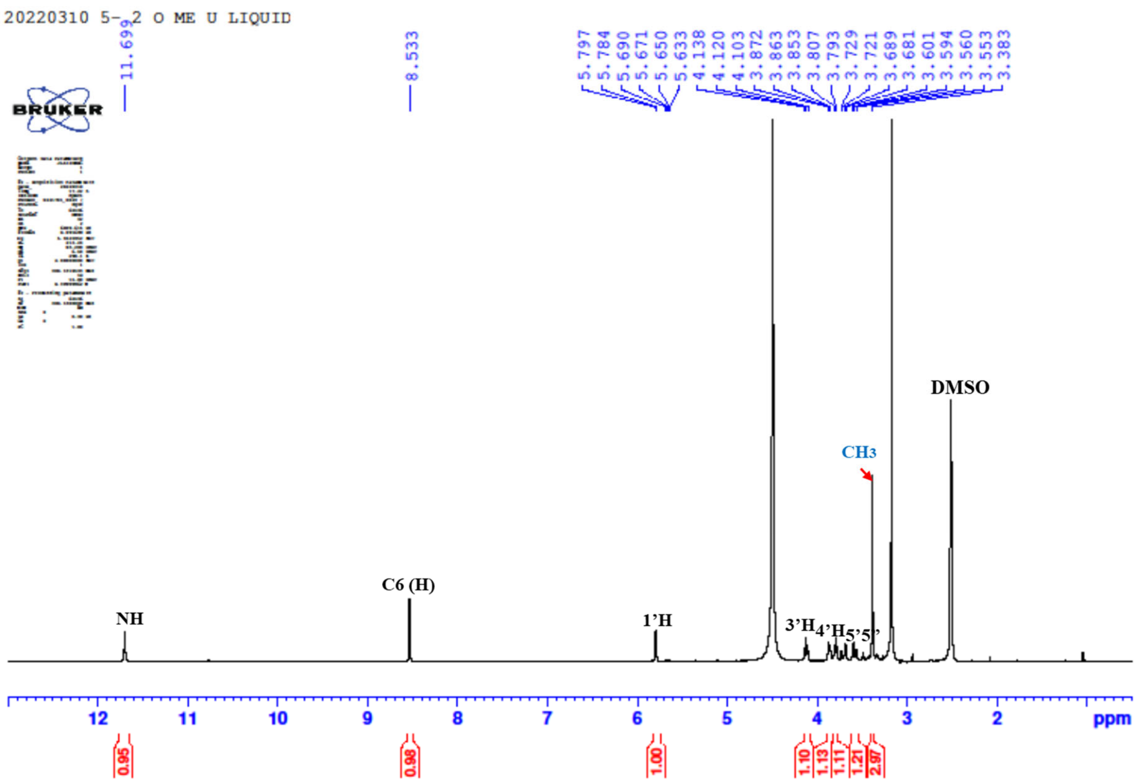
20220310 5-_2 O ME U LIQUID
11.699
8.533
5.797
5.784
5.690
5.671
5.650
5.633
4.138
4.120
4.103
3.872
3.863
3.853
3.807
3.793
3.729
3.721
3.689
3.681
3.601
3.594
3.560
3.553
3.383
NH
C6 (H)
1'H
3'H
4'H
5'5'
CH3
DMSO
12
11
10
9
8
7
6
5
4
3
2
ppm
0.95
0.98
1.00
1.10
1.13
1.11
1.21
2.97

(C)

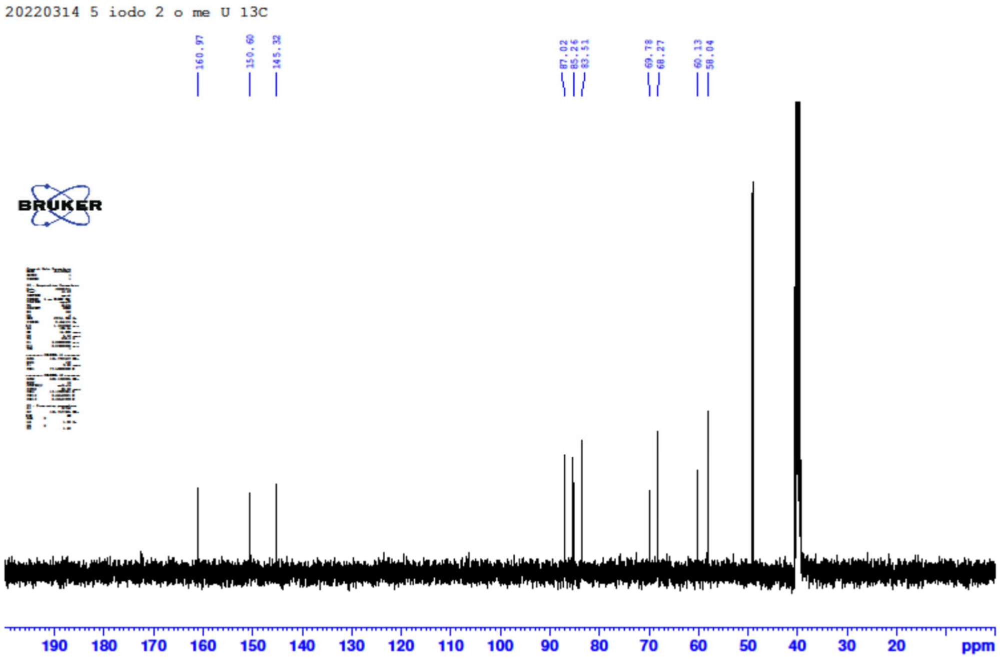

(D)

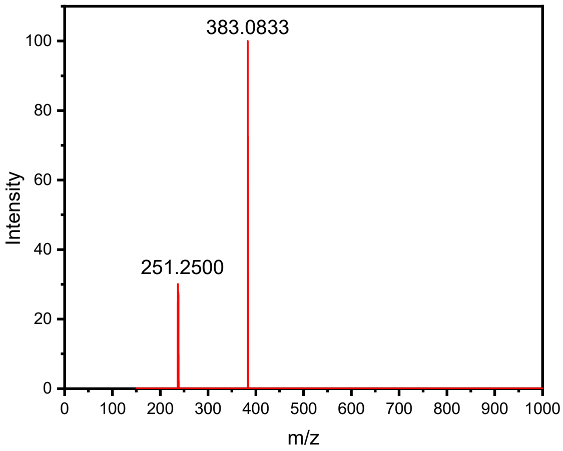

Figure S4. (A) Chemical structure and (B) ^{1}H NMR, (C) ^{13}C NMR, and (D) ESI-Mass spectra of 5-iodo-2′-OMe-uridine (5I-mU).

(A)

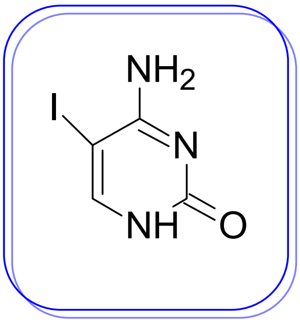

NH2
I
N
NH
O

(B)

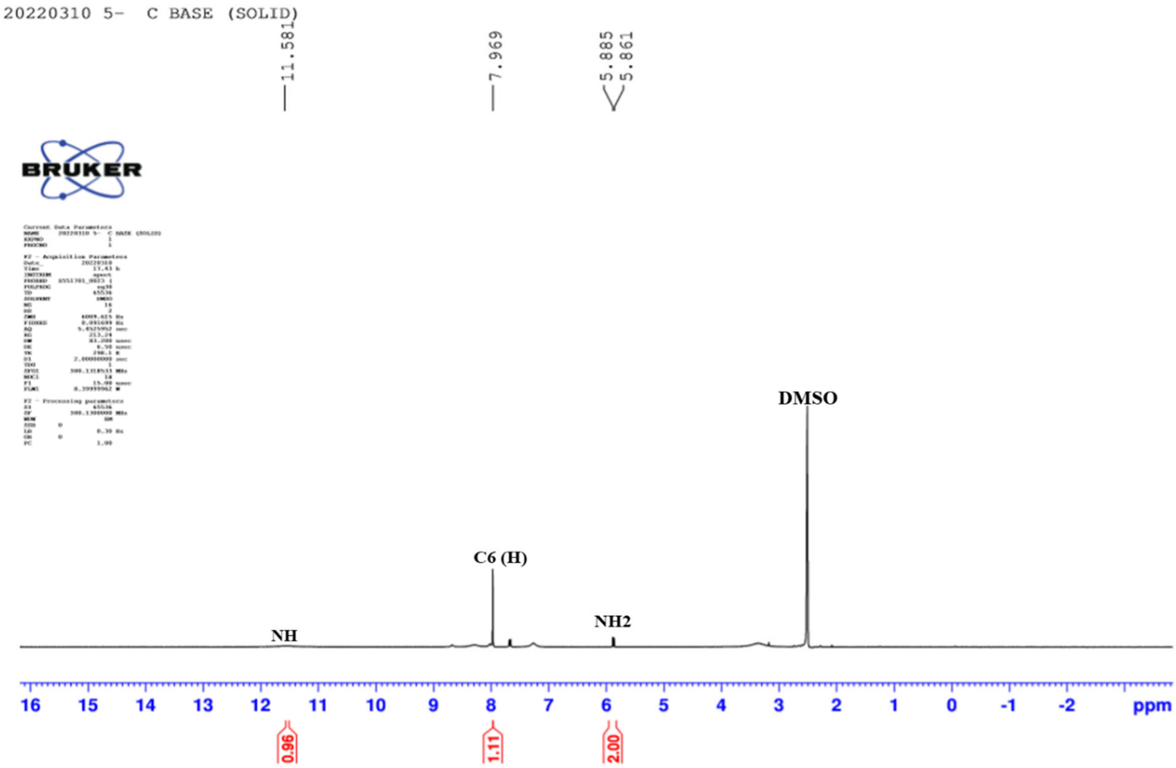

20220310 5- C BASE (SOLID)
11.581
7.969
5.885
5.861
DMSO
C6 (H)
NH2
NH
16
15
14
13
12
11
10
9
8
7
6
5
4
3
2
1
0
-1
-2
ppm
0.96
1.11
2.00

(C)

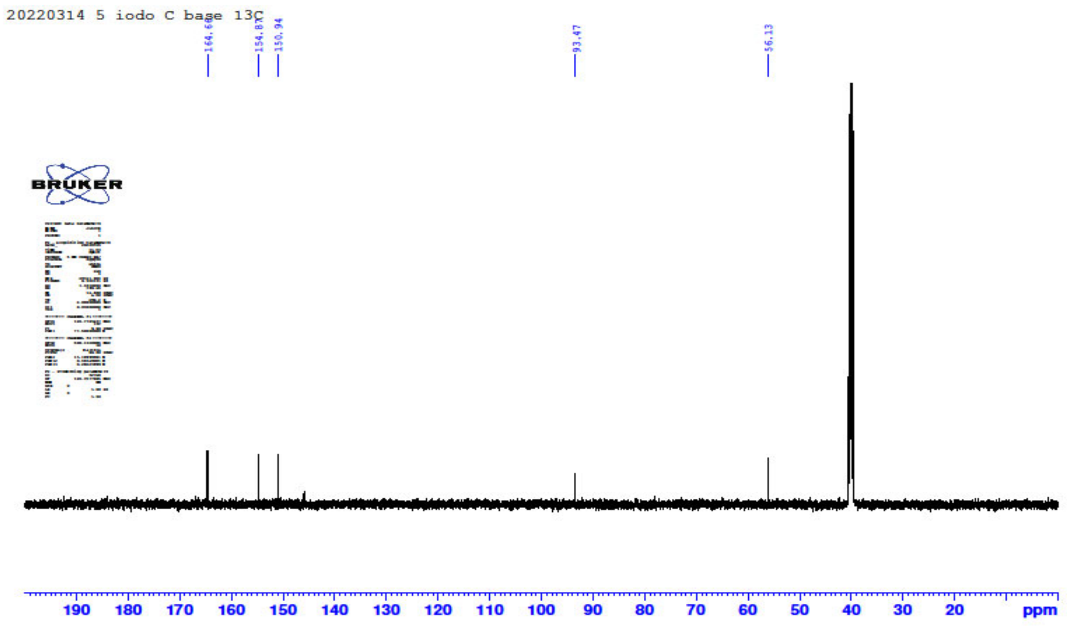

(D)

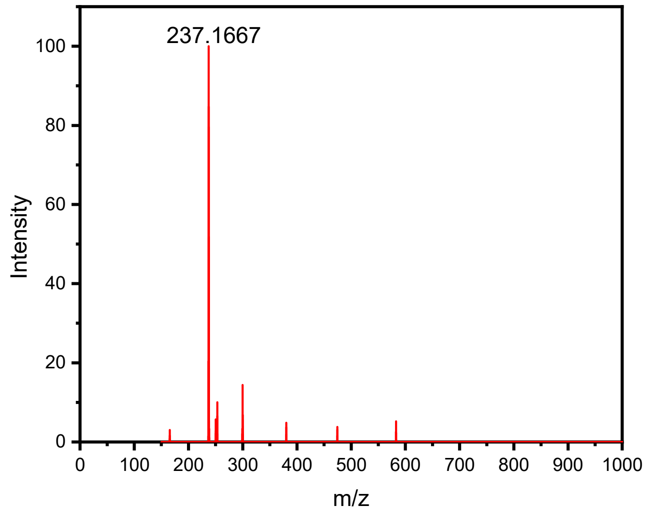

Figure S5. (A) Chemical structure and (B) ^{1}H NMR, (C) ^{13}C NMR, and (D) ESI-Mass spectra of 5-iodo-cytosine (5I-C).

(A)

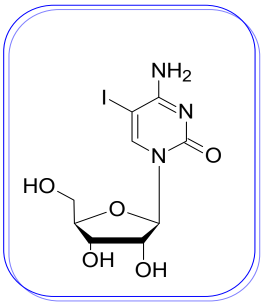
NH2
I
N
N
O
HO
O
OH
OH

(B)

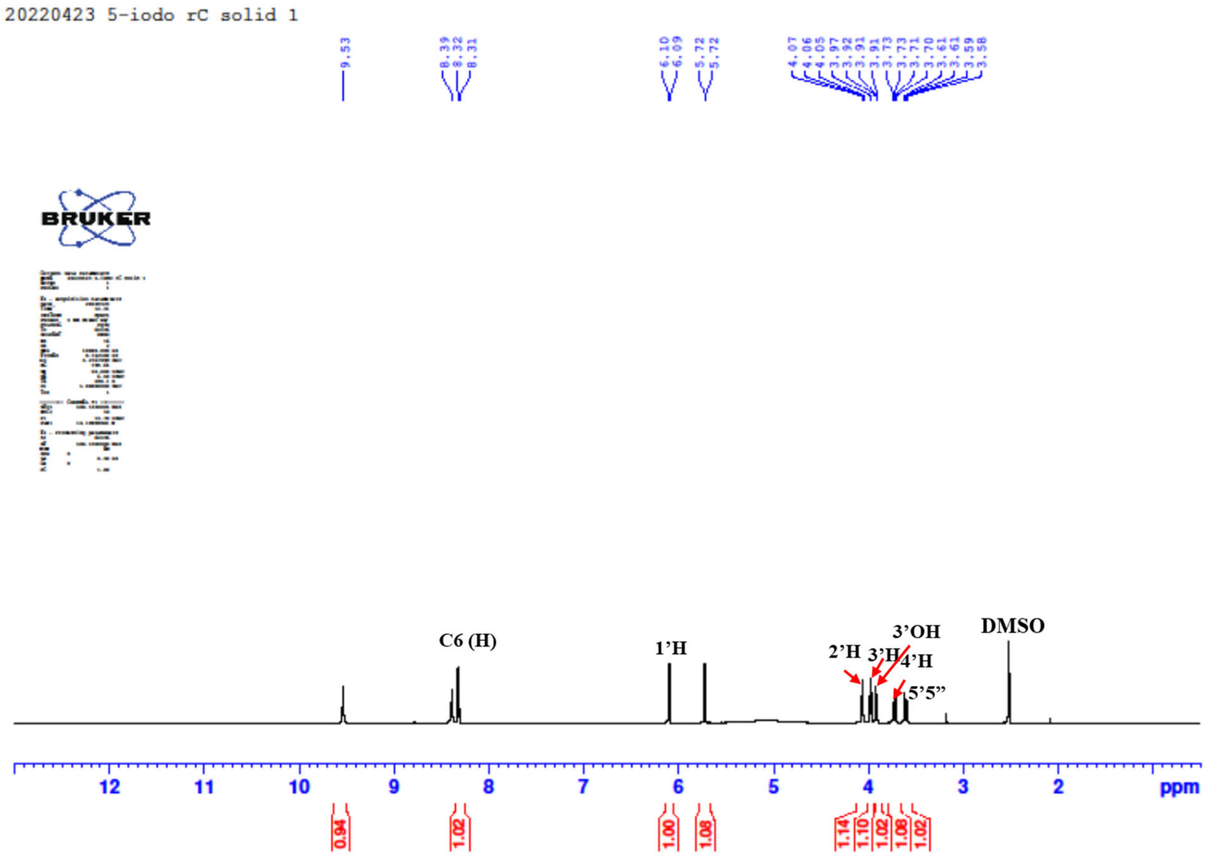
20220423 5-iodo rC solid 1
9.53
8.39
8.32
8.31
6.10
6.09
5.72
5.72
4.07
4.06
4.05
3.97
3.92
3.91
3.91
3.73
3.73
3.71
3.70
3.61
3.61
3.59
3.58
BRUKER
C6 (H)
1'H
2'H
3'H
3'OH
4'H
5'5"
DMSO
12
11
10
9
8
7
6
5
4
3
2
ppm
0.94
1.02
1.00
1.08
1.14
1.10
1.02
1.08
1.02

(C)

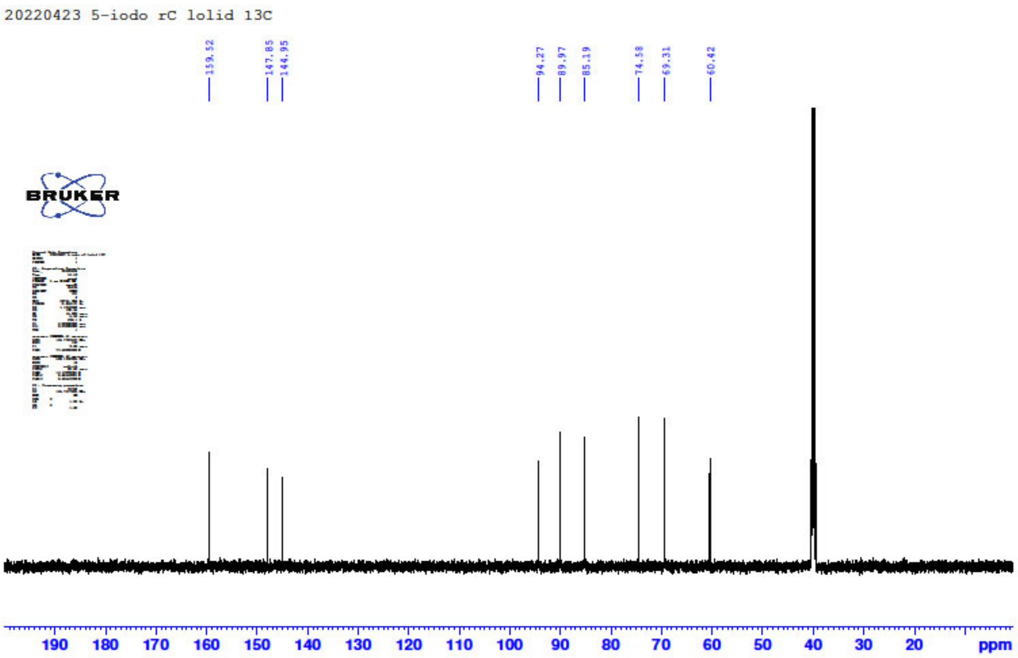

(D)

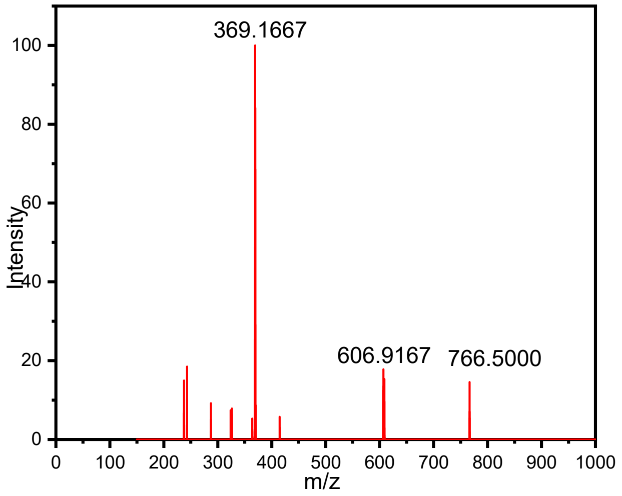

Figure S6. (A) Chemical structure and (B) ^{1}H NMR, (C) ^{13}C NMR, and (D) ESI-Mass spectra of 5-iodo-cytidine (5I-rC).

(A)

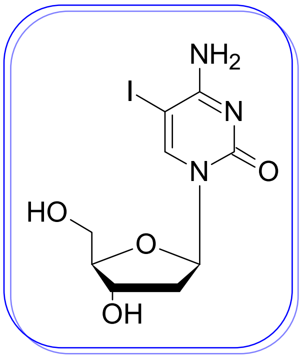
NH2
I
N
N
O
HO
O
OH

(B)

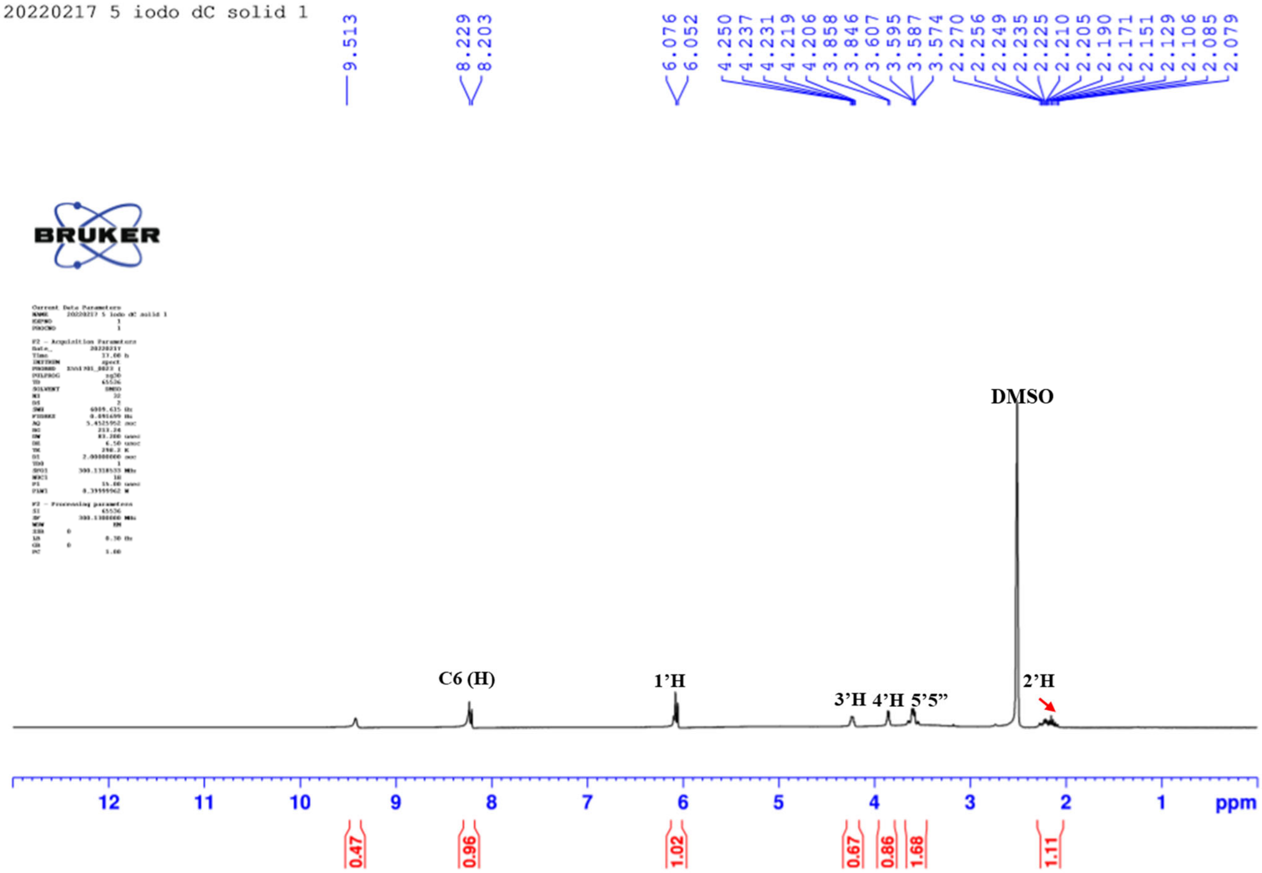
20220217 5 iodo dC solid 1
9.513
8.229
8.203
6.076
6.052
4.250
4.237
4.231
4.219
4.206
3.858
3.846
3.607
3.595
3.587
3.574
2.270
2.256
2.249
2.235
2.225
2.210
2.205
2.190
2.171
2.151
2.129
2.106
2.085
2.079
BRUKER
DMSO
C6 (H)
1'H
3'H 4'H 5'5"
2'H
12
11
10
9
8
7
6
5
4
3
2
1
ppm
0.47
0.96
1.02
0.67
0.86
1.68
1.11

(C)

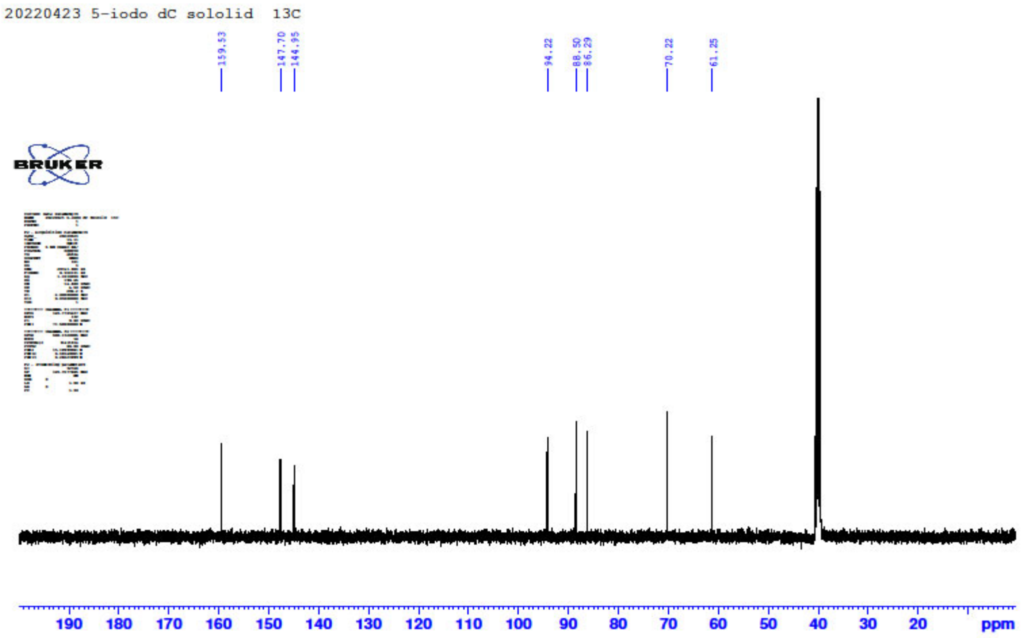

(D)

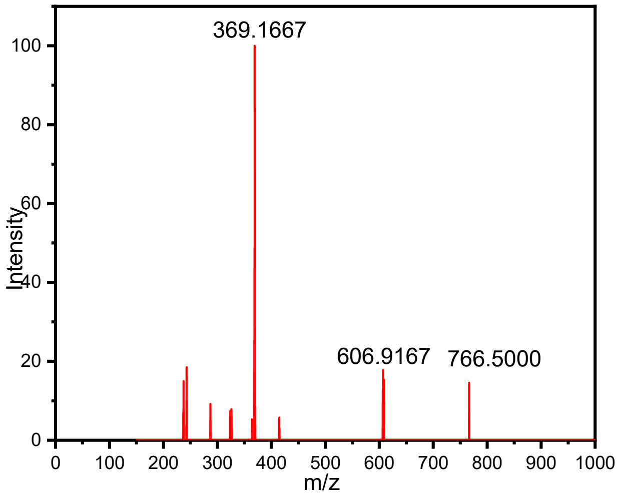

Figure S7. (A) Chemical structure and (B) ^{1}H NMR, (C) ^{13}C NMR, and (D) ESI-Mass spectra of 5-iodo-2'-deoxycytidine (5I-dC).

(A)

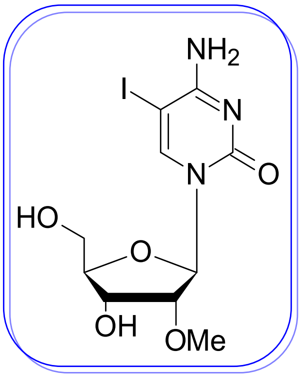
NH2
I
N
O
N
HO
O
OH
OMe

(B)

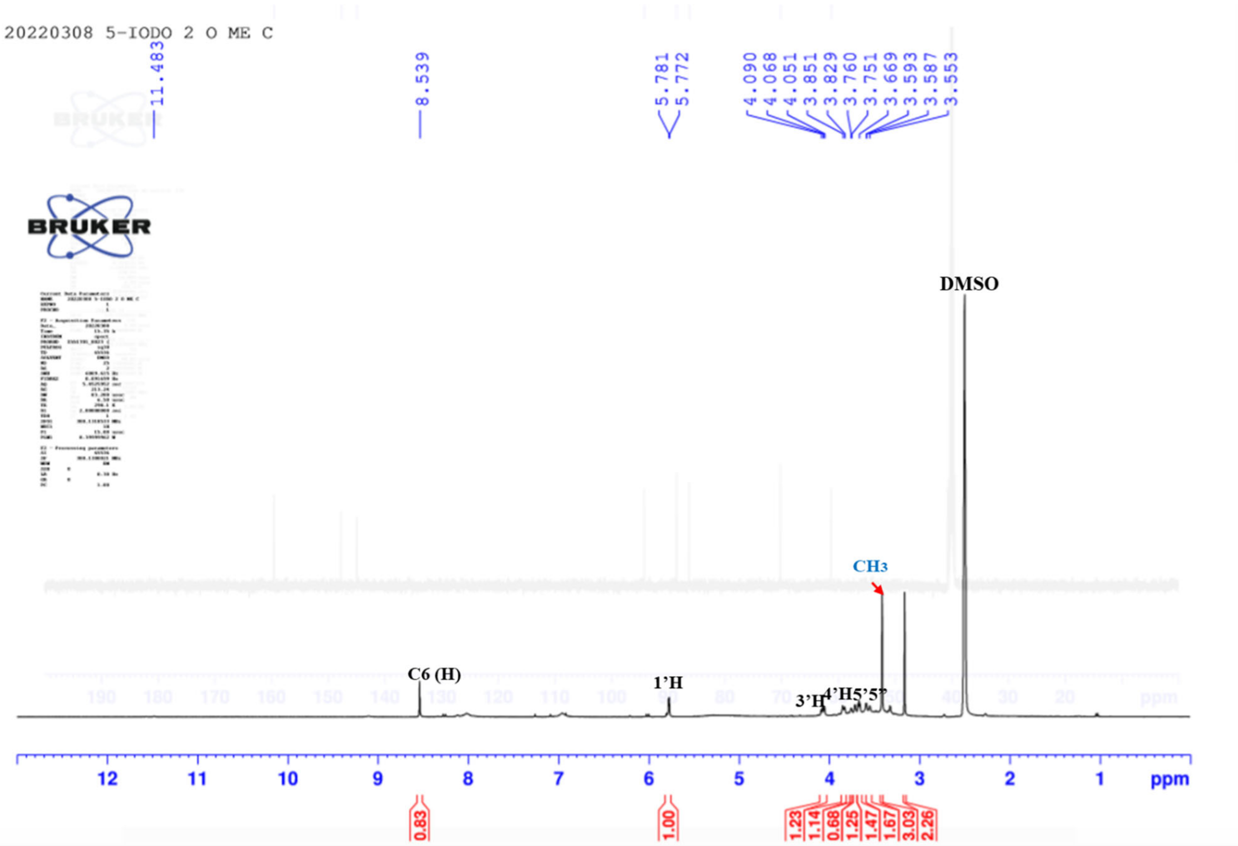
20220308 5-IODO 2 O ME C
11.483
8.539
5.781
5.772
4.090
4.068
4.051
3.851
3.829
3.760
3.751
3.669
3.593
3.587
3.553
BRUKER
DMSO
CH3
C6 (H)
1'H
3'H
4'H5'5"
12
11
10
9
8
7
6
5
4
3
2
1
ppm
0.83
1.00
1.23
1.14
0.68
1.25
1.47
1.67
3.03
2.26

(C)

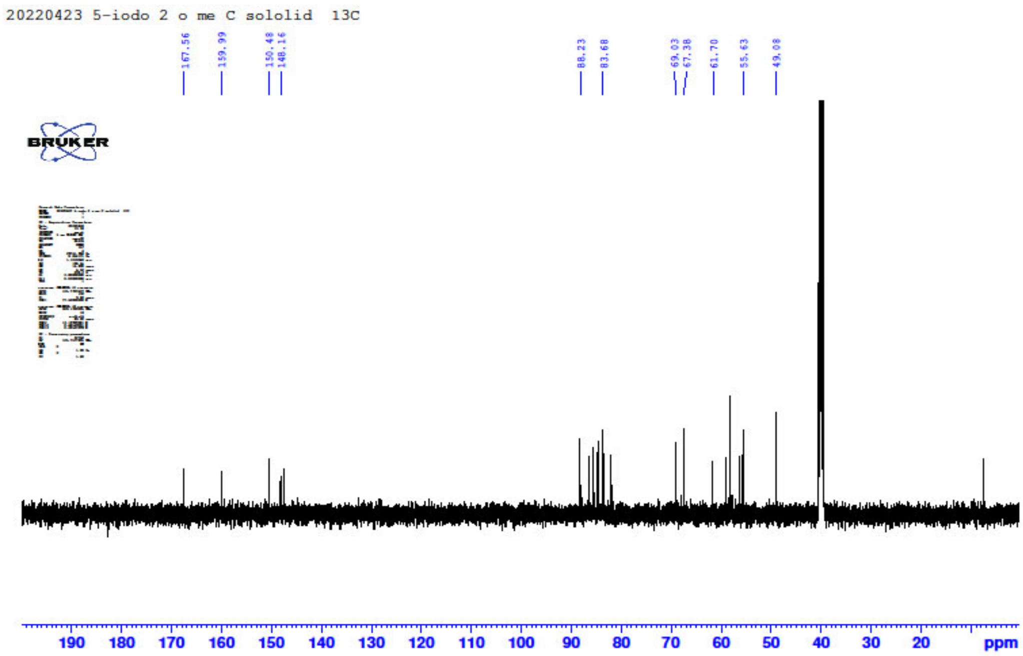

(D)

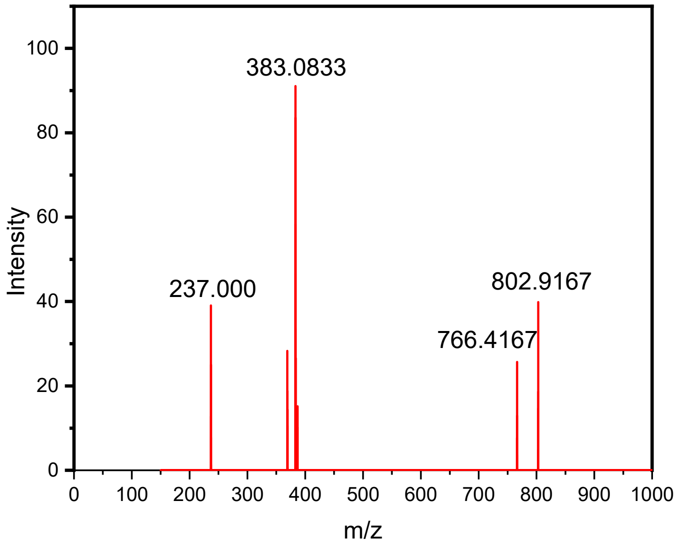

Figure S8. (A) Chemical structure and (B) ^{1}H NMR, (C) ^{13}C NMR, and (D) ESI-Mass spectra of 5-iodo-2′-OMe-cytidine (5I-mC).

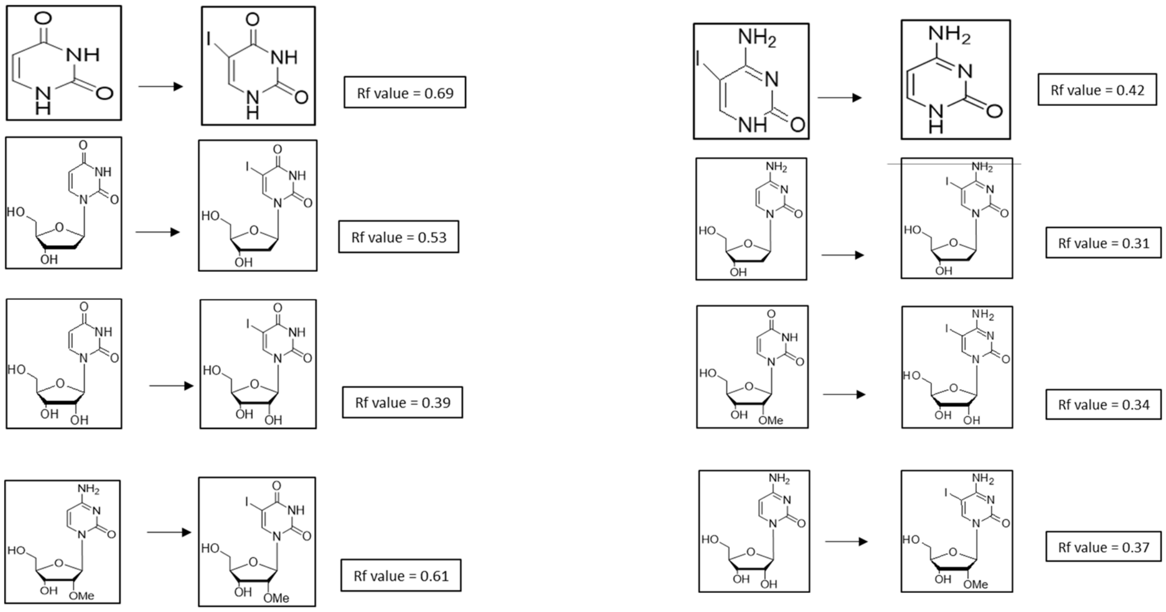

Figure S9. The TLC Rf values of fractions of 5-iodo pyrimidine derivatives (solvent CH_2Cl_2:MeOH (5:1)).
